# Echocontrast cystosonography versus micturating cystourethrography in the detection of vesicoureteric reflux

**DOI:** 10.2349/biij.7.1.e7

**Published:** 2011-01-01

**Authors:** MZ Faizah, Y Kanaheswari, CR Thambidorai, MA Zulfiqar

**Affiliations:** 1 Department of Radiology, Universiti Kebangsaan Malaysia Medical Center, Kuala Lumpur, Malaysia; 2 Department of Paediatrics, Universiti Kebangsaan Malaysia Medical Center, Kuala Lumpur, Malaysia; 3 Department of Surgery, Universiti Kebangsaan Malaysia Medical Center, Kuala Lumpur, Malaysia

**Keywords:** Echocontrast cystosonography, micturating cystourethrography, vesicoureteric reflux, sensitivity, specificity

## Abstract

**Purpose:**

To compare echocontrast cystosonography (ECS) using in-vivo agitated saline with fluoroscopic micturating cystourethrography (MCU) in the detection and grading of vesicoureteric reflux (VUR).

**Materials and methods:**

This was a prospective study of 25 children, who had MCU between 2007 and 2009. ECS was performed and findings documented prior to MCU. Baseline renal and bladder sonograms were obtained. The bladder was filled with normal saline followed by introduction of 10–20 mls of air to generate microbubbles. Detection of VUR was based on two sonographic criteria: (1) presence of microbubbles in the pelvicaliceal system (PCS), and (2) increase in dilatation of the PCS. VUR was graded as (1) Grade I: microbubbles seen in ureter only; (2) Grade II: microbubbles seen in non-dilated PCS; and (3) Grade III-V: microbubbles seen in dilated PCS. The ECS findings were compared using MCU as the gold standard.

**Results:**

Of the 50 kidney-ureter (K-U) units studied, ECS detected 9 of 10 K-U units with VUR on MCU. ECS did not detect a Grade II VUR. The sensitivity, specificity, accuracy, positive predictive value and negative predictive value for criterion 1 was 90%, 87.5%, 88%, 64.3% and 97%, respectively, compared to criterion 2 which was 70%, 90%, 86%, 64% and 92%, respectively. The grading of VUR was similar on both ECS and MCU except for one case.

**Conclusion:**

ECS using agitated saline was a sensitive technique for the detection of VUR. ECS grading was comparable with MCU grading of VUR.

## INTRODUCTION

Vesicoureteric reflux (VUR) is defined as retrograde flow of urine from the bladder into the renal collecting system that provides a pathway for ascent of bacteria in the presence of urinary tract infection [1–3]. This will result in renal scarring and subsequent renal hypertension and even worse, renal failure [4]. Early diagnosis and treatment of this problem will reduce the above risks and ensure the quality of life of children with VUR [5]. The current gold standard in diagnosing VUR is using micturating cystouretherography (MCU) [6–8]. This examination exposes the young patient to ionising radiation. The average effective dose for an MCU involving fluoroscopy and three radiographs is between 0.3 and 0.4 mSv [9].

In children with VUR, there is increasing concern over radiation exposure, especially in follow-up cases after conservative management or surgical intervention [9]. The use of ultrasound to detect VUR is an attractive option because it is tolerable to young patients and is a non-ionising modality. Ultrasound by itself is neither sensitive nor specific in the diagnosis of VUR [2]. However, the use of echo-contrast such as agitated saline or galactose solution during ultrasound has proven to be promising in the diagnosis of VUR [3, 8, 10]. A study done in 2001 concluded that the number of MCUs performed was significantly reduced (by almost 53%) as a result of the implementation of echocontrast cystosonography (ECS) as part of routine diagnostic imaging for VUR [11], thus reducing the radiation burden [6, 11]. The purpose of this study is to evaluate the effectiveness of ECS compared to MCU in the detection and grading of VUR.

## MATERIALS AND METHODS

### Patient population and study setting

This was a prospective study conducted between September 2007 and June 2009 in the Department of Radiology of a tertiary hospital. All paediatric patients (aged below 12 years) who were scheduled for micturating cystourethrography (MCU) for the assessment of VUR in the Radiology Department were included in this study. Verbal or written consent from the children’s parents or guardian was obtained.

Cases where both ECS and MCU could not be performed on the same day were excluded from this study. Other exclusion criteria included recent urinary tract infection (less than 6 weeks after last negative urine culture), no verbal or written consent obtained from the parents or caregiver, child did not turn up for the MCU procedure, and the doctor performing the ECS had prior knowledge of the child’s diagnosis before performing the ECS. There were 52 cases scheduled for MCU during the study period but only 33 cases came for the procedure and 25 patients were included in this study. This comprised of 15 boys and 10 girls aged from 1 month to 8 years, with mean age of 20 months.

The clinical indications for the study included recurrent urinary tract infection (n = 9), antenatal hydroureteronephrosis (n = 10), sacral agenesis with neurogenic bladder (n = 2), acute urinary retention (n = 1) and follow-up examinations after a course of conservative or surgical management (n = 3). None of the patients had solitary kidney. Therefore, a total of 50 kidney-ureter (K-U) units were evaluated for reflux. The study was approved by the Ethics Committee of the hospital.

### Data collection

Participating children were seen at the Paediatric Day Care centre for the insertion of the urinary catheter under aseptic technique and for administration of prophylactic stat dose of intramuscular (IM) gentamicin (2 mg/kg) by the paediatric medical officer. The child was then sent to the Radiology Department for the ECS and MCU. No sedation was given for the procedures.

ECS was performed using either Phillips IU-22 or Philips HD-11 ultrasound machine. The ultrasound machines were equipped with C5-2MHz convex and L12-5MHz linear transducers. The selection of ultrasound probe (either C5-2MHz convex or 12-5MHz linear transducer) was made based on the child’s body habitus. Basically, the L12-5MHz linear probe was used for the smaller child, aged below 2 years, and older children were imaged with the C5-2MHz curved probe.

The child was in a supine position throughout the procedure. Except when the ureter was obscured by overlying bowel gas, the child was positioned in supine oblique or prone position. After emptying the bladder via the pre-inserted urinary catheter, baseline ultrasound was performed to assess renal size and parenchymal echogenicity, and for the presence of pelvicaliceal and/or ureteric dilatation. Saline was then slowly instilled into the bladder via the urinary catheter using a 20 ml syringe until the amount reached the estimated age-related maximum bladder volume. The age-related maximum bladder volume was estimated as: volume (millilitres) = [age (in years) + 2] × 30 ml [12].

Microbubbles were generated by the introduction of 5–10 ml of air into the bladder with moderate force over 5–8 seconds. The observation for reflux was done immediately following administration of intravesical microbubbles. The distal ureter, the renal pelvis and the proximal ureter were observed intermittently (similar to intermittent screening in MCU) for about 3–5 minutes. Images were documented. For detecting Grade I reflux, the fluid-filled bladder was used as an acoustic window to image the distal ureters and therefore bowel gas was not a problem. The renal pelvis was imaged in two planes (longitudinal and transverse) to ensure that any echogenic foci observed were bubbles and not artifacts. In view of the instability of the microbubbles which normally underwent gradual dissolution, a second introduction of 5–10 ml of air was done to evaluate the opposite side. Assessment of the urethra was not consistently done during ECS as some of the children were restless during the procedure.

The two sonographic criteria used for the detection of VUR were: (1) real-time visualisation of moving microbubbles within the ureter and/or the PCS during introduction of air; and (2) increase in dilatation of the ureter and/or the PCS. VUR was graded as (1) Grade I: microbubbles seen in ureter only; (2) Grade II: microbubbles seen in non-dilated PCS; and (3) Grade III-V: microbubbles seen in dilated PCS.

Images were documented in copy films as well as in our PACS (Medweb) system. Documentation of the sonographic findings was done prior to MCU to avoid bias.The bladder was then emptied via the catheter and the child proceeded to the fluoroscopy room.

MCU was performed using the digital fluoroscopy system; Toshiba KXO-80G, Tokyo, Japan. It was performed by different radiology trainees who were blinded to the ECS result. Using the institution’s standard operating procedure (SOP), the child was positioned in supine position (during filling and full bladder) and then in the right anterior oblique as well as left anterior oblique positions (during voiding in order to visualise the urethra in profile). The MCU was performed using room temperature saline mixed with contrast agent (Ultravist® 300 mg/ml), to obtain 30% concentration. The urinary catheter was connected to a three-way tap, which was connected to the bag of diluted contrast placed 100cm above the level of the fluoroscopy table. The contrast was then instilled slowly (approximately 1 drop/second) into the urinary bladder. Total estimated bladder volume was also based on the previously mentioned formula. Intermittent screening of the urinary bladder was done during filling, full bladder and voiding. The urinary catheter was removed at the end of the procedure. The findings were documented with an EPS package software and Paxport-Agfa capture box. VUR seen on MCU was graded according to the International Reflux Study Committee Classification [13]. Images were recorded and stored as hardcopy films or saved in PACS system (Medweb) via DICOM DBOX6000 image viewer system.

### Data analysis

The two sonographic criteria for VUR were compared separately with MCU, and their sensitivity, specificity, accuracy, positive predictive value and negative predictive values were calculated.

## RESULTS

### ECS detection of VUR

A total of 50 kidney-ureter (K-U) units were evaluated. There was no immediate adverse reaction during the procedures. Of the 50 K-U units, 14 refluxing systems were demonstrated on ECS. Of these, 9 were confirmed to have VUR on MCU and 5 were false positive. MCU detected VUR in 10 K-U units. ECS did not detect a Grade II VUR (Table 1).

**Table 1:** Correlation between VUR seen on ECS and MCU

**Reflux on ECS**	**Reflux on MCU**		**Total**
	**Yes**	**No**	
Yes	9	5	14
No	1	35	36
Total	10	40	50

Using criterion 1, the sensitivity, specificity, accuracy, positive predictive value and negative predictive value for detecting VUR when compared with MCU was 90%, 87.5%, 88%, 64.3% and 97%, respectively (Table 2). Using criterion 2, the sensitivity, specificity, accuracy, positive predictive value and negative predictive value in detecting VUR was 70%, 90%, 86%, 64% and 92%, respectively (Table 2).

**Table 2:** Comparison between sonographic criteria for reflux on ECS and MCU

**Sonographic criteria for reflux on ECS**		**Reflux on MCU**	
		**Present (n=10)**	**Absent (n=40)**
Presence of microbubbles	Present	9	5
	Absent	1	35
Increased dilatation of PCS	Present	7	4
	Absent	3	36

**Figure 1:**
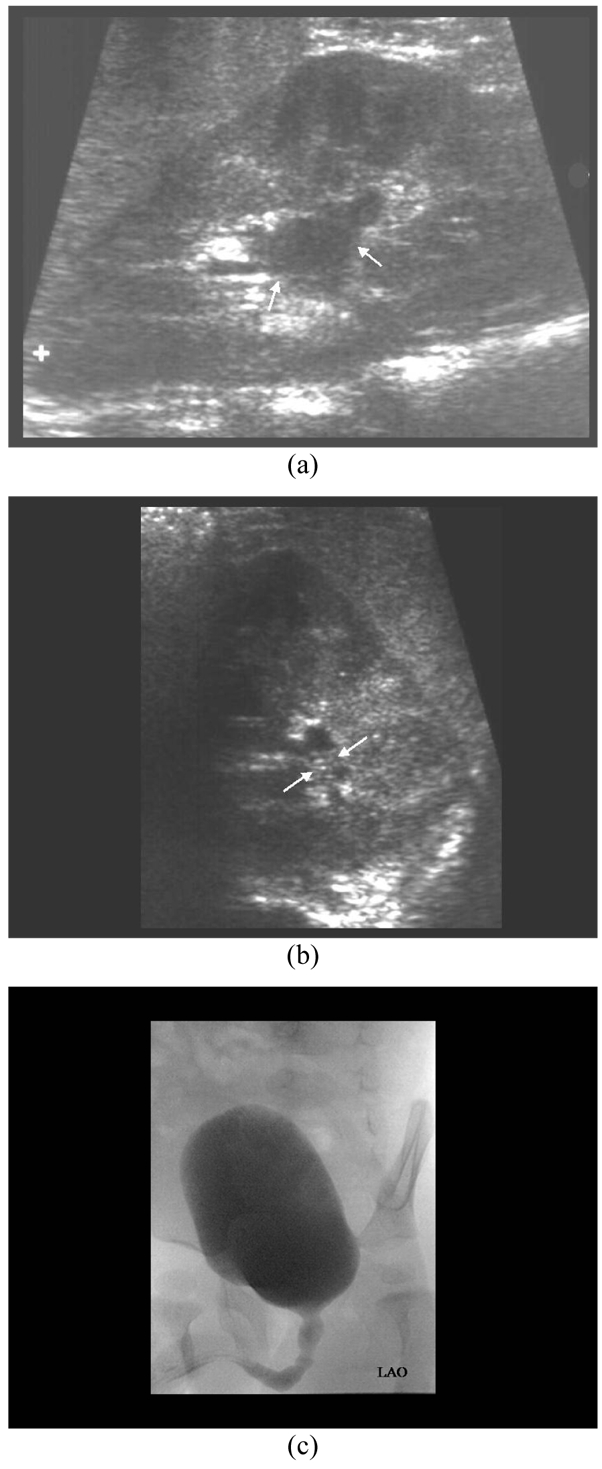
An eighteen months-old boy had history of urinary tract infection and positive urine culture for E.coli; a) Renal sonogram shows left mild hydronephrosis (arrows); b) ECS (in axial plane) demonstrates presence of microbubbles (arrows) in the dilated PCS without increase in PCS dilatation; c) MCU is normal.

**Figure 2:**
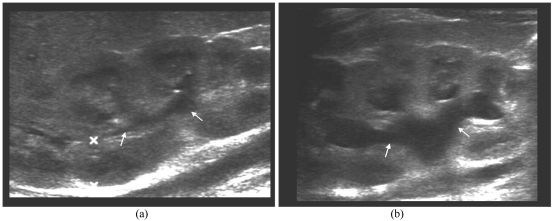
A one month-old child had history of antenatal bilateral hydronephrosis. a) Renal sonogram shows mild separation of right renal sinus (arrows); b) ECS shows increased dilatation of right renal pelvis (arrows) without presence of microbubbles. The MCU was normal.

**Figure 3:**
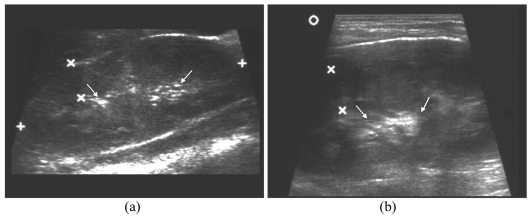
A six year-old girl had history of three urinary tract infections. a) Right renal sonogram shows compact sinus echoes (arrows); b) ECS shows no evidence of microbubbles and no PCS dilatation (arrows). The MCU showed right Grade II VUR.

Both criteria had false positive results (Figure 1 & 2). One case had no evidence of microbubbles in the PCS, and showed no PCS dilatation but had Grade II reflux on MCU (Figure 3). This was the only false negative result common for both ECS criteria. There were three false negative results using criterion 2.

### ECS Grading of VUR

**Figure 4:**
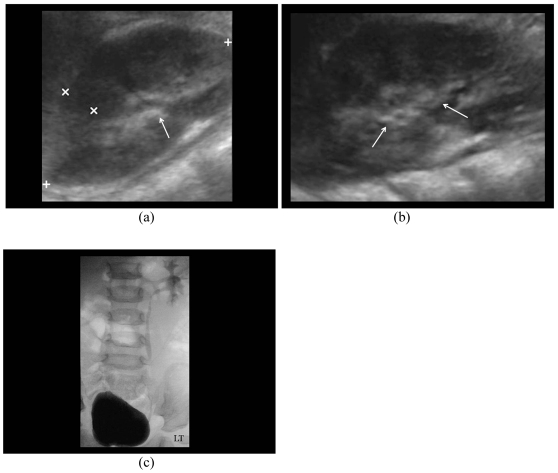
A sixteen months-old girl had one episode of E.coli urinary tract infection. a) Sonogram shows compact sinus echoes (arrow); b) ECS shows air microbubbles in left PCS (arrows) which is not dilated; c) MCU demonstrates left Grade II VUR.

**Figure 5:**
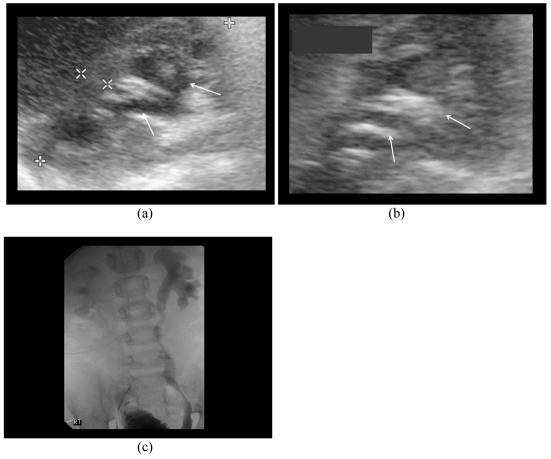
A seven years-old boy had spina bifida and neurogenic bladder. a) The sonogram shows left mild to moderate hydronephrosis (arrows) with thinning of renal cortex; b) ECS demonstrates presence of microbubbles in the dilated PCS and increased dilatation of the PCS (arrows); c) MCU shows left Grade IV VUR.

**Figure 6:**
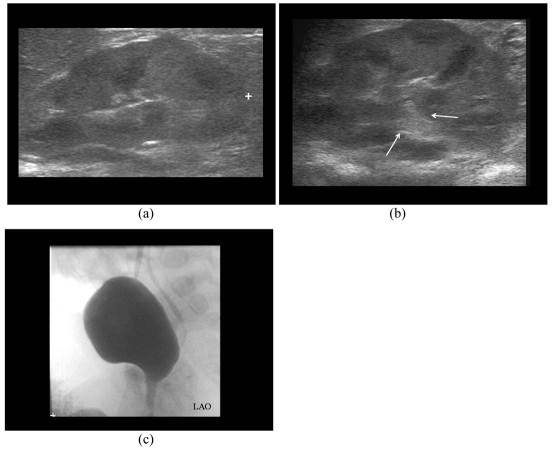
A four months-old girl who had E. coli urinary tract infection. a) Right sonogram shows the PCS is not dilated; b) ECS shows presence of microbubbles in dilated right PCS (arrows); c) MCU demonstrates bilateral Grade II VUR (arrows). The left Grade II VUR was seen as presence of microbubbles in non-dilated PCS on ECS.

Three K-U units with microbubbles in non-dilated PCS proved to have Grade II VUR on MCU (Figure 4). Of the 6 K-U with microbubbles in dilated PCS, 5 had either Grade III, IV or V (Figure 5) and one had Grade II VUR on MCU (Figure 6). ECS had correctly graded 75% Grade II, and 100% Grade III, IV and V VUR (Table 3). There was no Grade I VUR on either ECS or MCU.

**Table 3:** Distribution of K-U units based on location of microbubbles on ECS as compared to VUR Grading on MCU

**Location of microbubbles & ECS Grading of VUR**	**MCU Grading of VUR**				
	**I**	**II**	**III**	**IV**	**V**
Ureter only, Grade I					
Non-dilated PCS, Grade II		3			
Dilated PCS, Grade III-V		1	2	1	2

## DISCUSSION

### ECS detection of VUR

MCU and radionuclide imaging (DMSA) have been used for the diagnosis of VUR [14–18]. In view of the presence of radiation burden in these two imaging procedures, ECS is a promising alternative for detection of VUR [16]. ECS has an additional advantage of enabling continuous observation without radiation exposure [8, 16]. Considering the high diagnostic agreement [3, 14–16] between ECS and MCU, the latter up to now having been considered the gold standard for VUR diagnosis, it was decided that ECS should be performed. In the authors’ first experience of using ECS for detecting VUR, agitated saline had been used.

The technique of ECS is operator- and patient-dependent [18]. As in many new procedures, the evaluation of VUR using ECS involves a learning curve, particularly in lower grades of VUR [18]. An excessively restless and moving patient may cause a decrease in accuracy [18]. Sedation was not given to reduce the distress of the children who had to undergoing both ECS and MCU; therefore it was difficult to perform real-time assessment of the PCS in the restless subjects and this might have contributed to false negative results.

The instability of microbubbles is well recognised [8]. The method of producing in-vivo microbubbles [8] was used, rather than producing in-vitro microbubbles [10] to overcome the problem of bubble instability. This method seemed to be adequate to generate bubbles, at least in the bladder, but the stability of the microbubbles refluxing into the K-U unit has not yet been established. Variable stability of the microbubbles was observed despite standardising the temperature of the saline and method of administration of the microbubbles. However, another factor that could contribute to the stability of the microbubbles could be the child’s bladder volume, which may require administration of more microbubbles in bigger childen as compared to children with smaller body habitus. Instability of the microbubbles may also contribute to the false negative results.

The sensitivity and specificity of ECS in detecting VUR in this study were comparable with other reports. Two other studies using agitated saline as the echocontrast media showed better sensitivity and specificity compared to this study [8, 10]. However, more promising results were described in studies using galactose-based contrast agents [14, 15–18]. A recent study reported the results of ECS as follows: sensitivity 57–100%, specificity 85–100%, diagnostic accuracy 78–96% and positive/negative predictive values 58–100%/87–100%, respectively [17].

There were two criteria evaluated in this study. Comparing between these two criteria, criterion 1 showed better sensitivity than criterion 2. This result was similar to a previous study which concluded that visualisation of moving microbubbles within the collecting system was a definitive sonographic sign of reflux [8]. Criterion 2 showed slightly better specificity than criterion 1.

Review of the images of false positive results using criterion 1 revealed that, in some cases, the apparent presence of microbubbles was probably due to ultrasound artefacts. However, in other cases, the presence of microbubbles in the PCS could not be disregarded. Continuous observation might make ECS better than MCU because of the intermittent nature of VUR [16]. Some authors have discussed the probability that false positive cases were actually true positive based on positive findings on radionuclide imaging [3, 8, 10, 15]. However, radionuclide imaging was not done for any of the false positive cases in this study.

False positive results using criterion 2 could be due to a full bladder. A distended bladder in a patient able to void normally could cause dilatation of the collecting system [19]. This problem was overcomed by imaging the pelvicaliceal system after the bladder was emptied. When the dilatation persisted, a positive diagnosis of reflux was made.

### ECS Grading of VUR

Of ten VUR detected on MCU, nine cases were comparable for grading of VUR as one case was a false negative case (a Grade II VUR). One case, which was documented as Grade II VUR on MCU, showed presence of microbubbles in a dilated PCS. This is comparable to Grade III and above on ECS, which means it was overgraded by ECS. Otherwise, the rest of the grading of VUR on ECS was concordant with the grading of VUR on MCU.

Previous studies have shown that ECS tends to grade the VUR on a higher grade compared to MCU [3, 5, 15]. One study showed that ECS tended to depict a higher grade of VUR than did MCU when both procedures demonstrated VUR [3]. A recent study of comparative aggregated data for reflux grading between ECS and MCU indicated that: (a) reflux grades between the two methods are concordant in about 75% of PCS; (b) the discordant findings are primarily due to a significant number of Grade I reflux episodes on MCU being Grade II or higher on ECS [17].

Several authors have proposed a different grading system for ECS. Some studies grade reflux according to the classification of the International Reflux Study Committee [3, 14–16]. One study using echogenic contrast media SH U508A [3] and a 5-level grading system similar to VUR grading in MCU concluded that ECS was comparable to MCU in VUR grading. A detailed reflux grading has been proposed. This study concluded that a reflux grading system, which is similar to the one used in MCU, can be applied in ECS [15].

There were several limitations to this study. Firstly, the technique is highly operator-dependent. The development of expertise involves a learning curve, which means more cases need to be done. Secondly, microbubbles produced by agitated saline were rather unstable and resulted in additional introduction of air when assessing the contralateral K-U unit. Commercial echogenic contrast media are currently available and have been proven to be stable enough to assess both K-U units without additional contrast administration.

The initial plan of study was to perform ECS during the filling as well as voiding phases of the bladder. However, since the non-sedated children were crying and restless, the assessment of the urethra was inconsistent. This is considered as one of the limitations of the study, as VUR was usually assessed during filling of urinary bladder, full bladder and voiding phase on MCU. The sub-optimal ECS was to reduce the child’s anxiety before being subjected to MCU immediately after. This problem could be overcome with oral sedation.

## CONCLUSION

ECS using agitated saline proved to be a sensitive imaging technique for the detection of VUR. It provided simultaneous evaluation of renal contours, size and parenchymal echogenicity; in addition to bladder visualisation. Both sonographic criteria were definitive signs of reflux and ‘presence of microbubbles in PCS’ was more sensitive but less specific than ‘increased dilatation of the PCS’. ECS grading of VUR proved to be comparable with MCU.
